# Prognostic Impact of 24-Hour Pulse Pressure Components in Treated Hypertensive Patients Older Than 65 Years

**DOI:** 10.3390/diagnostics13050845

**Published:** 2023-02-23

**Authors:** Francesca Coccina, Anna M. Pierdomenico, Chiara Cuccurullo, Jacopo Pizzicannella, Oriana Trubiani, Sante D. Pierdomenico

**Affiliations:** 1 Department of Innovative Technologies in Medicine & Dentistry, University “Gabriele d’Annunzio”, Chieti-Pescara, 66100 Chieti, Italy; 2 Department of Medicine and Aging Sciences, University “Gabriele d’Annunzio”, Chieti-Pescara, 66100 Chieti, Italy; 3 Department of Engineering and Geology, University “Gabriele d’Annunzio”, Chieti-Pescara, 66100 Chieti, Italy

**Keywords:** ambulatory blood pressure, hypertension, prognosis, pulse pressure

## Abstract

(1) Background: The aim of this study was to assess the prognostic impact of 24-hour pulse pressure (PP), elastic PP (elPP) and stiffening PP (stPP) in elderly treated hypertensive patients; (2) Methods: In this retrospective study, we evaluated 745 treated hypertensive subjects older than 65 years who underwent ambulatory blood pressure monitoring to assess 24-hour PP and 24-hour elPP and stPP, as calculated by a mathematical model. The association of these PP components with a combined endpoint of cardiovascular events was investigated; (3) Results: The 24-hour PP, elPP and stPP were 59 ± 12.5, 47.5 ± 9.5 and 11.5 ± 6.5 mmHg, respectively. During the follow-up (mean 8.4 years), 284 events occurred, including coronary events, stroke, heart failure hospitalization and peripheral revascularization. In the univariate Cox regression analysis, 24-hour PP, elPP and stPP were associated with the combined outcome. After the adjustment for covariates, per one standard deviation increase, 24-hour PP had a borderline association with risk (hazard ratio (HR) 1.16, 95% confidence interval (CI) 1.00–1.34), 24-hour elPP remained associated with cardiovascular events (HR 1.20, 95% CI 1.05–1.36) and 24-hour stPP lost its significance. (4) Conclusions: 24-hour elPP is a predictor of cardiovascular events in elderly treated hypertensive patients.

## 1. Introduction

Pulse pressure (PP) is one of the most studied blood pressure (BP) parameters, and it is calculated as the difference between systolic and diastolic BP. PP is representative of the response of the arterial system to the intermittent left ventricular (LV) ejection and is affected by cardiac factors, vascular resistance, arterial wall properties and pulse wave reflection [1,2]. PP becomes wider after 50 years of age [3,4]. Various studies have investigated the prognostic value of PP and have suggested that it predicts cardiovascular outcomes when detected by either clinic BP measurement [4,5,6,7,8,9], ambulatory BP monitoring [10,11,12,13,14,15,16,17,18,19,20,21] or home BP recording [22]. However, despite this classic definition of PP, findings on its prognostic relevance have not been consistent across studies [15,18]. PP has generally been considered a parameter of arterial stiffness, which globally describes the ratio between the pressure and volume incremental changes [23,24,25,26]. However, it has been reported that the relationship between volume and pressure is not linear in human arteries [23,24,25,26]. Indeed, after LV ejection, this relation presents a progressive upward curve with increasing pressure [23,24,25,26]. In such a context, it has recently been suggested that PP may be divided into two different components, one reflecting the first phase or arterial elasticity, which has been named elastic PP (elPP), and another reflecting the succeeding phase or the tendency of the arteries to stiffen under higher BP, which has been named stiffening PP (stPP) [23,24,25,26]. A simple mathematical model has been developed to calculate these components by 24-hour ambulatory BP monitoring data [24], as reported in the methods section, which is based on the assumption of an exponential pressure–volume relationship and a linear systolic–diastolic BP association. The investigation of the clinical relevance of these components is at its beginning, having been performed in only three studies [24,25,26]. The aforesaid studies [24,25,26], including different population types, reported interesting but conflicting results about the effect of these new PP components on clinical outcomes. Thus, other data could be helpful in adding further knowledge on this topic. As previously reported, PP increases with aging, and 65 years is the most frequent age adopted today to describe the transition to the elderly in Western countries. In this context, the aim of this study was to assess the prognostic impact of 24-hour PP, 24-hour elPP and 24-hour stPP in treated hypertensive patients older than 65 years. The primary outcome was to evaluate the impact of PP components on the occurrence of cardiovascular events, hypothesizing that the various components could have had a different influence on the cardiovascular outcome.

## 2. Materials and Methods

### 2.1. Patients

This study is a retrospective examination analyzing the new topic of PP components, among our prospectively collected data. We included 745 treated hypertensive patients older than 65 years, the most frequent age currently used to define the transition to the elderly status in Western nations, from a cohort of 2264 sequential treated individuals with complete follow-up. The global population was aged 30 to 90 years and was recruited from December 1992 to December 2012. All these patients had been referred to our hospital outpatient clinic for the evaluation of BP control. Among the initial population of 781 subjects older than 65 years, 36 were lost during the follow-up. For this specific study, the inclusion criteria were: (1) hypertensive patient; (2) use of antihypertensive drugs; (3) age older than 65 years. Subjects with secondary hypertension were excluded, as well as those with a recent cardiovascular event (6 months), an acute illness, dementia, cancer and a disability. All the subjects underwent clinical evaluation, an electrocardiogram, routine laboratory tests, an echocardiographic examination and non-invasive ambulatory BP monitoring at baseline. The study population came from the same geographical area (Chieti and Pescara, Abruzzo, Italy). Our prospective observational study was approved by the Institutional Review Committee in 1992. For this specific manuscript, ethical approval was waived due to the retrospective nature of the study. Subjects gave written informed consent to be included in the study and for anonymous data processing.

### 2.2. Clinic BP Measurement

Clinic BP was detected by a physician using a mercury sphygmomanometer and appropriate-sized cuffs. Measurements were performed three times in a quiet room, 2 minutes apart, after at least 5 min of rest, and the mean value was used as the BP for the visit. Clinic systolic BP < 140 mmHg and clinic diastolic BP < 90 mmHg were defined as normal. As a consequence, clinic systolic BP ≥ 140 mmHg and/or clinic diastolic BP ≥ 90 mmHg were defined as hypertension.

### 2.3. Ambulatory BP Monitoring

Ambulatory BP monitoring was performed with a noninvasive recorder (SpaceLabs 90207, Redmond, WA, USA) within 1 week of the clinical evaluation. The technical aspects have been previously reported [27]. We evaluated the following ambulatory BP parameters from the examination carried out at baseline: daytime (while awake, as reported in the diary), nighttime (while asleep, as reported in the diary), 24-hour systolic and diastolic BP, 24-hour systolic and diastolic BP standard deviation (SD) and 24-hour heart rate. We calculated the 24-hour PP as the difference between 24-hour systolic and diastolic BP and the 24-hour mean arterial pressure (MAP) as 24-hour diastolic BP + 24-hour PP/3. The components of PP were determined from ambulatory BP data, as previously reported [24]. Briefly, the following five-step procedure was performed: (1) calculation of an individual 24-hour PP; (2) calculation of the ratio between the standard deviation of 24-hour systolic and diastolic BP, named K; (3) calculation of the “widening factor” (WIF), expressed as (K − 1)/ln(K) − 1; (4) calculation of elPP as PP/(1 + WIF); (5) calculation of stPP as PP − elPP. The recordings of all the subjects were of good quality (at least 70% of valid readings during the 24-hour period, at least 20 valid readings while awake, with at least two valid readings per hour, and at least 7 valid readings while asleep, with at least one valid reading per hour), in line with the European Society of Hypertension requirements [28].

### 2.4. Echocardiography

A comprehensive echocardiographic examination, including two-dimensional, M-mode, doppler and color doppler assessment, was performed. The measurements of the left atrial (LA) and LV dimensions and the calculation of the LV mass were made according to standardized methods [29]. The LA diameter (cm) was indexed by body surface area (m^2^), and LA enlargement was defined as LA diameter/body surface area ≥ 2.4 cm/m^2^ [29]. The LV mass value was indexed by height^2^.^7^, and LV hypertrophy was defined as LV mass/height^2^.^7^ >50 g/m^2^.^7^ in men and >47 g/m^2^.^7^ in women [30]. The LV ejection fraction (EF) was computed using the Teichholz formula or the Simpson rule [29] and was defined as low when it was <50%.

### 2.5. Follow-Up

The included individuals were followed up in our outpatient clinic or by their general practitioner. The occurrence of events was recorded during follow-up visits or by telephone interviews of the general practitioner, the patient or a family member, followed by a visit if the patient was alive. Medical records were obtained to confirm the events. We evaluated a combined endpoint of cardiovascular events including coronary events (sudden death, fatal and nonfatal myocardial infarction and coronary revascularization), fatal and nonfatal stroke, heart failure hospitalization and peripheral revascularization. Only the first event during follow-up was analyzed. Outcomes were defined according to standard criteria, as previously reported [31,32,33,34,35]. In this study, the investigator (F.C) who evaluated the combined endpoint was blinded to the BP data.

### 2.6. Statistical Analysis

The data are reported as the means ± standard deviation, the median (interquartile range) or as numbers and percentages. Pearson’s correlation was used. The event rate is expressed as the number of events per 100 patient years based on the ratio of the observed number of events to the total number of patient years of exposure up to the terminating event or censor. Univariate Cox regression analysis was used to assess the association of patients’ characteristics and clinic PP, 24-hour PP, 24-hour elPP, 24-hour stPP and 24-hour MAP with outcome. Multivariate Cox regression analyses were used to evaluate whether clinic PP, 24-hour PP, 24-hour elPP, 24-hour stPP and 24-hour MAP were independently associated with cardiovascular risk. In the multivariate analyses, as detailed later, we included variables that were significantly associated with the outcome in the univariate analysis, that is, age, diabetes mellitus, previous cardiovascular events, estimated glomerular filtration rate, LV hypertrophy, LA enlargement and asymptomatic LV systolic dysfunction. Moreover, we decided a priori to include the following covariates in the multivariate models, regardless of their association with risk: gender (because of the potential differences in PP between men and women), 24-hour heart rate (because of its reported potential impact on PP), 24-hour MAP (to account for the steady component of BP beyond the pulsatile component represented by PP) and classes of antihypertensive drugs used (because of their potentially different influence on afterload, preload, cardiac contractility, stroke volume and, possibly, arterial wall properties). We built a model including the other variables and 24-hour PP and a model including the other variables and 24-hour elPP and 24-hour stPP. The unadjusted and adjusted hazard ratio (HR) and the 95% confidence interval (CI) for cardiovascular events of clinic PP, 24-hour PP, 24-hour elPP, 24-hour stPP and 24-hour MAP are reported per 1 SD increase in each parameter. We used the forced entry model in the multivariate Cox regression analysis. Statistical significance was defined as *p* < 0.05. Analyses were conducted with the SPSS 21 software package (SPSS Inc. Chicago, IL, USA).

## 3. Results

The characteristics of the study population are reported in Table 1. The median age was 71 years (interquartile range 68–76 years), and 41% were men. Only a minority of patients had a previous event, including stroke (*n* = 32), myocardial infarction (*n* = 23), heart failure hospitalization (*n* = 16) and peripheral revascularization (*n* = 4).

The clinic and ambulatory BP data are reported in Table 2. The 24-hour stPP was about 20% of the 24-hour PP.

**Figure 1:**
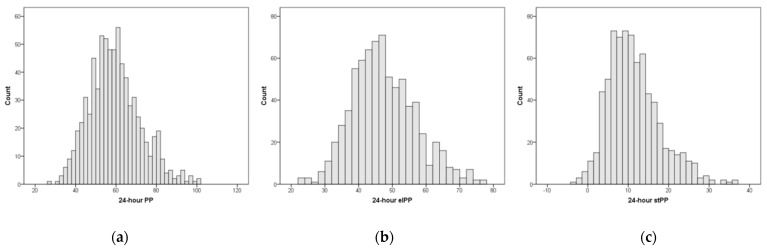
Distribution of 24-hour pulse pressure (PP) (**a**), elastic PP (elPP) (**b**) and stiffening PP (stPP) (**c**) in the study population.

**Figure 2:**
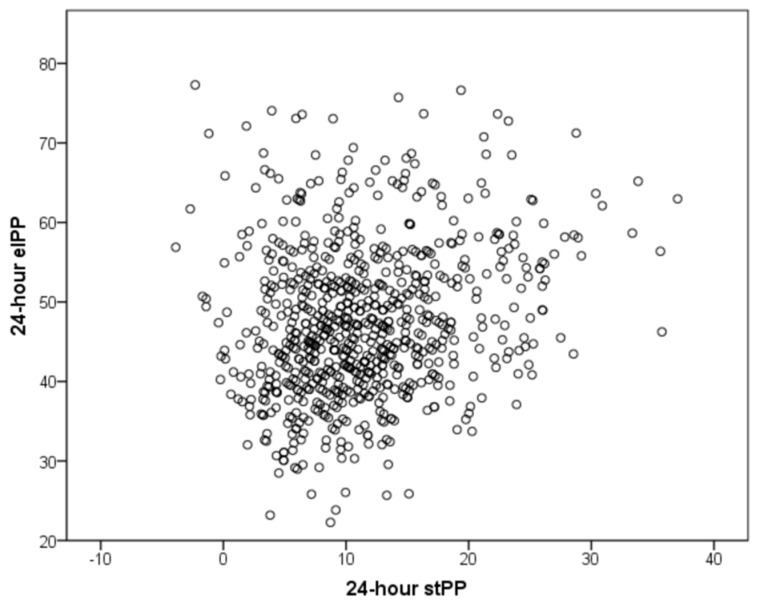
Correlation between 24-hour elastic pulse pressure (elPP) and stiffening pulse pressure (stPP) in the study population.

The distribution of 24-hour PP, elPP and stPP is reported in Figure 1, and the correlation between 24-hour elPP and stPP is shown in Figure 2. Distributions appear to be nearly Gaussian, the skewness and kurtosis being 0.49 and 0.20 for 24-hour PP, respectively, 0.41 and 0.12 for 24-hour elPP, respectively, and 0.79 and 0.81 for 24-hour stPP, respectively. The 24-hour elPP and 24-hour stPP were significantly (*p* < 0.001) but weakly correlated (r = 0.21).

The antihypertensive therapy of the study population is reported in Table 3. Diuretics and angiotensin-converting enzyme inhibitors were the most frequently used drug classes. Double therapy was the most frequent strategy.

During the follow-up (8.4 ± 4.8 years, range 0.4–20.5), 284 events occurred. The event-rate was 4.52 per 100 patient years. Specifically, there were 86 coronary events, 110 cerebrovascular events, 82 heart failure events requiring hospitalization and 6 peripheral revascularizations.

The results of the univariate Cox regression analysis are reported in Table 4. Among the characteristics reported in Table 1, age, diabetes, previous events, estimated glomerular filtration rate, LV hypertrophy, LA enlargement and asymptomatic LV systolic dysfunction were associated with outcome. The investigated BP parameters, that is, clinic PP, 24-hour PP components and 24-hour MAP, were all associated with outcome.

**Figure 3:**
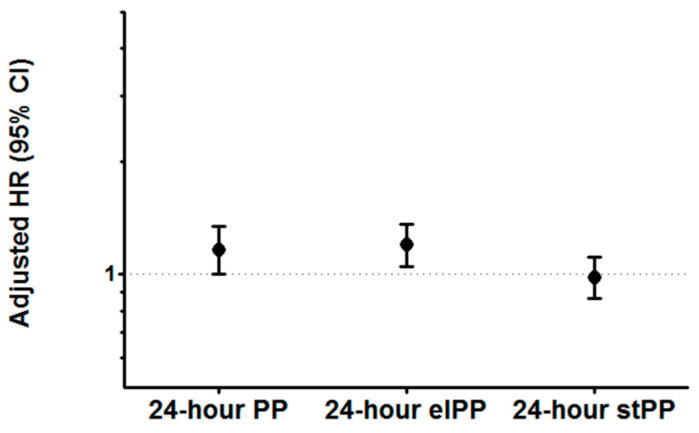
Adjusted hazard ratio (HR) and 95% confidence interval (CI) for cardiovascular events of 24-hour pulse pressure (PP), elastic PP (elPP) and stiffening PP (stPP), expressed per one standard deviation increment of each PP component. Adjustment included age, sex, diabetes, previous events, estimated glomerular filtration rate, left ventricular (LV) hypertrophy, left atrial enlargement, asymptomatic LV systolic dysfunction, 24-hour heart rate, 24-hour mean arterial pressure and the use of a diuretic, beta blocker, calcium channel blocker, angiotensin-converting enzyme inhibitor, angiotensin receptor blocker and alpha blocker. Adjusted HR (95% CI) is 1.16 (1.00–1.34), 1.20 (1.05–1.36) and 0.98 (0.86–1.11) for 24-hour PP, 24-hour elPP and 24-hour stPP, respectively.

After the adjustment for various covariates in the Cox multivariate analysis, clinic PP lost its significance (HR (95% CI) 1.03 (0.89–1.20)). In the same type of analysis, 24-hour MAP remained associated with an increased risk in both the model including 24-hour PP and that including 24-hour elPP and 24-hour stPP (HR (95% CI) per 1 SD increment 1.26 (1.10–1.45) and 1.23 (1.07–1.42), respectively). The results of the multivariate Cox regression analyses regarding 24-hour PP components are reported in Figure 3. After the adjustment for covariates, 24-hour PP had a borderline association with risk (HR (95% CI) 1.16 (1.00–1.34) per 1 SD increment), 24-hour elPP remained significantly associated with cardiovascular outcome (HR (95% CI) 1.20 (1.05–1.36) per 1 SD increment) and 24-hour stPP lost its significance.

When the 24-hour elPP and 24-hour stPP were analyzed in separate Cox models in the multivariate analysis, the results were the same.

## 4. Discussion

This study shows that 24-hour elPP is a predictor of cardiovascular outcome, suggesting that it may help to describe the risk profile in the specifically studied population of elderly treated hypertensive patients.

At present, there are few studies on this topic in the literature [24,25,26]. Gavish and Bursztyn have been the pioneers of this new conceptualization of 24-hour PP, developing a model-based method to divide 24-hour PP into two components, that is, 24-hour elPP and 24-hour stPP [24]. They investigated the predictive power of these PP components for all-cause deaths in 1999 subjects of a mean age 56 years, of whom 60% had treated hypertension [24]. During a 5-year follow-up, 5.1% of the patients died. The prediction of all-cause deaths was observed only in patients with a heart rate less than 70 beats/min. An increase in risk by 48% and 58% per 1 SD increment of 24-hour PP and 24-hour stPP, respectively, was reported. The 24-hour elPP was not associated with risk. None of the PP variables had predictive power for the higher-heart-rate subgroup. In another study, Bursztyn et al. [25] evaluated the aforementioned 24-hour PP components and their impact on outcome in a population of 1745 individuals without a history of cardiovascular disease, with a mean age of 61.4 years. During the follow-up, 21% of the subjects died from non-cardiovascular causes, 12% died from cardiovascular causes and 17% had a stroke. After the adjustment for various covariates, 24-hour PP, elPP and stPP tended to be associated with total mortality, cardiovascular mortality and stroke morbidity. However, among individuals with a median heart rate lower than 68.5 beats/min, total and cardiovascular mortality were predicted by 24-hour elPP (HR (95% CI) 1.23 (1.08–1.40) and 1.29 (1.07–1.57), respectively), and in the subgroup of treated hypertensive patients, these outcomes were even better-predicted. Stroke morbidity was not predicted by either 24-hour PP or 24-hour PP components. Gavish et al. [26] further analyzed the prognostic impact of 24-hour PP components in the mixed population of the International Database on Ambulatory Blood Pressure in Relation to Cardiovascular Outcomes (IDACO). During the follow-up, among patients aged 60–70 years, 22% had a cardiovascular event, and among those aged more than 70 years, 48.5% experienced an event. In patients aged 60–70 years and in those aged more than 70 years, the HR (95% CI) for cardiovascular events per 1 SD increase in 24-hour PP was 1.28 (1.16–1.41) and 1.14 (1.09–1.20), respectively. In the same age groups, the HR (95% CI) per 1 SD increase in 24-hour elPP was 1.27 (1.16–1.40) and 1.13 (1.08–1.19), and the HR (95% CI) per 1 SD increase in 24-hour stPP was 1.11 (1.00–1.23) and 1.06 (1.00–1.12). In the main analysis, the patients were not divided into those with lower or higher 24-hour heart rates [26]. When the 24-hour heart rate was taken into account in a sensitivity analysis (Supplementary File), the 24-hour PP and 24-hour elPP were associated with an increased risk in patients with both lower and higher heart rates in the age groups described above.

Our study shows similarities and differences in comparison with previous ones [24,25,26]. The similarities include the characteristics of the 24-hour PP components and their interrelationship. Some differences are present regarding the prognostic value of each 24-hour PP component. Gavish and Bursztyn [24] evaluated the impact of 24-hour PP components on total mortality and found that 24-hour PP and 24-hour stPP predicted the risk in patients with a lower heart rate. Bursztyn et al. [25] assessed the influence of these new PP components on total mortality, cardiovascular mortality and stroke and observed that 24-hour elPP predicted total and cardiovascular mortality among individuals with a lower heart rate. In the last study by Gavish et al. [26], when focusing on patients older than 60 years from a mixed population, it was found that 24-hour PP and 24-hour elPP were similarly associated with an increased risk of cardiovascular events, regardless of the 24-hour heart rate. In our study, which included only elderly treated hypertensive patients, we observed that 24-hour PP had a borderline association with outcome and that 24-hour elPP was significantly associated with an increased risk of cardiovascular events, even after the adjustment for various covariates including risk markers, 24-hour MAP, 24-hour heart rate and antihypertensive drug classes. In addition, we found that the prognostic value of 24-hour MAP was similar to that of 24-hour elPP. There may be several reasons for the discrepancies: (1) different ethnicities in the various studies; (2) different population types including normotensive subjects, untreated and/or treated hypertensive patients; (3) different age ranges of the studied populations; (4) different studied endpoints and (5) different adjustments in the multivariate Cox regression analysis.

Despite the potential discrepancies, however, our study and previous studies indicate that disentangling 24-hour PP into its components provide a new and clinically useful instrument for better understanding cardiovascular pathophysiology and for better stratifying the prognostic profile of hypertensive patients. Our data suggest that the elastic component dominates the association of 24-hour PP with risk in the elderly. It has been suggested that the interplay between elastin, collagen fibers and smooth muscle cells in the arterial wall may influence PP and its components, elastin being the main determinant of elPP and collagen fibers, together with increasing BP, the main determinant of stPP [23,24,25,26]. Aging, with or without potential concomitant diseases, may be associated with the degradation and fragmentation of the elastic fibers and an increase in collagen fibers [23,24,25,26]. These structural and functional modifications of the arterial wall impair global vascular stiffness and PP but probably have a major impact on the elPP component. This aspect could help explain our finding. The increase in elPP, which is representative of the increased stiffness during diastole and the first phase of systole, leads to reduced diastolic BP and increased systolic BP, which can affect cardiovascular outcomes. Whether a specific treatment could help delay damage to the elPP component from adulthood to old age or whether a specific treatment could improve it in the elderly may be a topic for future research.

The present study has some limitations. First, we studied only Italian patients, and our results may not be applicable to other ethnic groups. Second, we studied only treated hypertensive patients older than 65 years, and our data cannot be extrapolated to other types of hypertensive populations. Third, some of these patients were followed up in our outpatient clinic and some were followed up by their family doctors. Thus, BP control was not known in all the patients. Fourth, we did not perform an analysis by dividing the patients into those with a 24-hour heart rate below or above the median. Dividing the population into two groups would have reduced the number of events for each group, weakened the event-to-variable ratio in each model and weakened the statistical power. Thus, we analyzed the population as a whole and adjusted for the 24-hour heart rate in multivariate Cox models. Fifth, though our database was prospectively collected, this is a retrospective analysis about the new topic of PP components. Sixth, we did not specifically design a study for evaluating the risk associated with 24-hour PP components, but this study is part of a prospective assessment of the prognostic value of ambulatory BP parameters and other risk markers in our initially treated hypertensive patients.

Our study also has some strengths, including (1) a fairly large sample size, (2) the demonstration that the assessment of 24-hour PP components is easily applicable in a hypertensive population, (3) a large number of cardiovascular events and (4) the adjustment of risk by using a substantial number of covariates, including antihypertensive drug classes.

## 5. Conclusions

Our data show that 24-hour elPP is a predictor of cardiovascular outcomes in elderly treated hypertensive patients. Disentangling 24-hour PP into its components provides a new and useful instrument for better stratifying the prognostic profile of hypertensive patients.

## Figures and Tables

**Table 1:** Characteristics of the study population.

*n*.	745
Age, years	71 (68–76)
Men, *n* (%)	304 (41)
Body mass index, kg/m^2^	27.5 ± 4.0
Smokers, *n* (%)	73 (10)
Diabetes, *n* (%)	114 (15)
Previous events, *n* (%)	75 (10)
eGFR, mL/min	62 ± 14
LDL cholesterol, mg/dL	127 ± 29
LV hypertrophy, *n* (%)	245 (33)
LA enlargement, *n* (%)	168 (23)
ALVSD, *n* (%)	36 (5)

ALVSD, asymptomatic left ventricular systolic dysfunction, that is, those without symptoms and EF <50%; eGFR, estimated glomerular filtration rate; LDL, low-density lipoprotein; LA, left atrial; LV, left ventricular.

**Table 2:** Blood pressure and heart rate values of the study population.

*n*.	745
Clinic systolic BP, mmHg	152 ± 18
Clinic diastolic BP, mmHg	86 ± 10
Clinic PP, mmHg	66.0 ± 15.5
24-hour systolic BP, mmHg	133 ± 14
24-hour diastolic BP, mmHg	74 ± 8
24-hour PP, mmHg	59.0 ± 12.5
24-hour elastic PP, mmHg	47.5 ± 9.5
24-hour stiffening PP, mmHg	11.5 ± 6.5
24-hour MAP, mmHg	93 ± 9
24-hour systolic SD, mmHg	13.5 ± 3.0
24-hour diastolic SD, mmHg	9 ± 2
24-hour HR, beats/min	68 ± 9

BP, blood pressure; MAP, mean arterial pressure; PP, pulse pressure; SD, standard deviation.

**Table 3:** Antihypertensive therapy of the study population.

*n*.	745
Diuretic, *n* (%)	431 (58)
Beta blocker, *n* (%)	210 (28)
Calcium antagonist, *n* (%)	260 (35)
ACE-inhibitor, *n* (%)	396 (53)
ARB, *n* (%)	188 (25)
Alpha blocker, *n* (%)	104 (14)
Single therapy, *n* (%)	180 (24)
Double therapy, *n* (%)	344 (46)
Triple therapy, *n* (%)	176 (24)
Quadruple or more therapy, *n* (%)	45 (6)

ACE, angiotensin-converting enzyme; ARB, angiotensin receptor blocker.

**Table 4:** Results of the univariate analysis.

Parameter	HR (95% CI)
Age (10 years)	2.69 (2.17–3.44)
Diabetes (yes vs. no)	1.82 (1.31–2.52)
Previous events (yes vs. no)	1.51 (1.00–2.30)
eGFR (10 mL/min)	0.86 (0.80–0.93)
LV hypertrophy (yes vs. no)	1.95 (1.55–2.47)
LA enlargement (yes vs. no)	1.46 (1.14–1.88)
ALVSD (yes vs. no)	2.65 (1.73–4.04)
Clinic PP (1 SD)	1.28 (1.13–1.45)
24-hour PP (1 SD)	1.51 (1.37–1.67)
24-hour elPP (1 SD)	1.46 (1.33–1.60)
24-hour stPP (1 SD)	1.23 (1.10–1.38)
24-hour MAP (1 SD)	1.31 (1.18–1.46)

ALVSD, asymptomatic left ventricular systolic dysfunction; CI, confidence interval; eGFR, estimated glomerular filtration rate; elPP, elastic pulse pressure; HR, hazard ratio; LA, left atrial; LV, left ventricular; MAP, mean arterial pressure; PP, pulse pressure; SD, standard deviation; stPP, stiffening pulse pressure; 1 SD is 15.5, 12.5, 9.5, 6.5 and 9 mmHg for clinic PP, 24-hour PP, 24-hour elPP, 24-hour stPP and 24-hour MAP, respectively.

## Data Availability

The data underlying this article are available on reasonable request from the corresponding author.
